# A comparison of interferential current efficacy in elderly intervertebral disc degeneration patients with or without sarcopenia: a retrospective study

**DOI:** 10.1186/s12891-024-07337-w

**Published:** 2024-03-13

**Authors:** Hui Yuan, Lini Dong, Ou Zhang, Xiaoxiao Wang, Zejun Chen, Yunchao Li, Haoyu He, Guohua Lü, Jing Li, Lei Kuang

**Affiliations:** 1 Department of Spinal Surgery, The Second Xiangya Hospital of Central South University, Changsha, Hunan Province 410001 P.R. China; 2 Department of Geriatrics, The Second Xiangya Hospital of Central South University, Changsha, Hunan Province 410001 P.R. China; 3 Medical Education and Microbiology, California University of Science and Medicine, 1501 Violet Street, Colton, CA 92324 USA

**Keywords:** Sarcopenia, Intervertebral disc degeneration, Paravertebral muscles, Bioelectrical impedance analysis, Interferential current

## Abstract

**Background:**

Intervertebral disc degeneration and sarcopenia are both age-related diseases without effective treatments. Their comorbidities may worsen the prognosis, and further studies on interaction and therapy are needed. The purpose of the study was to investigate the prevalence of sarcopenia in intervertebral disc degeneration, and to compare the characteristics of intervertebral disc degeneration with and without sarcopenia and effects of interferential current.

**Methods:**

One hundred twenty disc degeneration patients were included from 2021 to 2022 in a single institute. Medical records, examination results and radiological reports were reviewed. Patients with sarcopenia were screened and grouped according to Asian Working Group for Sarcopenia 2019. VAS, ODI, SARC-F, SMI, gait speed (GS), grip strength, disc Pfirrmann grading, standard cross-sectional area (SCSA), degree of fatty infiltration (DFF), and nerve conduction velocity (NCV) were assessed before and after treatment.

**Results:**

The prevalence of sarcopenia in intervertebral disc degeneration was 28.3%. The difference of VAS, ODI, disc Pfirrmann grading, SCSA, DFF and NCV between two groups were significant before intervention (*P* < 0.05), SCSA and DFF were related to the degree of disc degeneration. The improvement of SMI, GS, grip strength, VAS, SARC-F and ODI in intervertebral disc degeneration with sarcopenia group was significant after intervention, as well as SMI, GS, grip strength, VAS and ODI in those without sarcopenia (*P* < 0.05). The improvement of grip strength, GS, ODI and SARC-F in intervertebral disc degeneration with sarcopenia group were greater than the one without sarcopenia (*P* < 0.05), whereas there was no significance in improvement degree of other indicators between the two groups (*P* > 0.05).

**Conclusion:**

The prevalence of sarcopenia was high in intervertebral disc degeneration, and paravertebral muscles degeneration correlated with the degree of disc degeneration. Compared to those without sarcopenia, intervertebral disc degeneration patients with sarcopenia have more severe pain, poorer mobility and neurological function. Interferential current is effective in intervertebral disc degeneration patients and sarcopenia patients.

## Introduction

Intervertebral disc degeneration (IDD) is a typical and frequently-occurring disease including disc herniation, and spondylolisthesis. It is considered to be a main contributor to lower back pain (LBP), numbness and lower-limb dysfunction, which has become a serious medical problem and imposing a financial and health burden to the individual and society for its high morbidity [1, 2]. Sarcopenia is an age-related disease that refers to the loss of muscle mass, strength and function, which may increase the risk of falls, fractures, and body dysfunction [3–5]. Currently, there are no approved therapies as the etiology of the two diseases are still not clearly defined. Previous studies found the prevalence of sarcopenia is 10–20% in the elderly and up to 25% in the lumbar spinal stenosis (LSS) [6–8]. The comorbidity of the two aggravates the condition and worsen the prognosis. The quality of life in lumbar disease patients with sarcopenia is not only affected, but also the risk of falls, the prevalence of osteoporosis, the rate of complications and re-admission increases, as well as a potentially prolonged hospitalization and even death [9–11]. Evidence suggests that IDD patients and patients with muscle atrophy or skeletal muscle dysfunction achieved good results after interferential currents (IFC) electrical stimulation [12–16]. It is a medium-frequency alternating current with amplitude-modulated in low frequency proposed by Austrian physicist Nemec in the 1950s [17], which can produce similar physiological effects to low-frequency current. As an applied electrical stimulation for the management of musculoskeletal diseases, it can reduce skin impedance to reach deep muscle tissue without increasing the patient's discomfort, and is effective in increasing neuromuscular excitability, relieving inflammation, eliminating edema, reducing nerve or muscle pain, promoting nerve regeneration, enhancing muscle strength, and improving physical mobility [18–20]. Additionally, interferential current stimulation is simple to use, safe, and has fewer adverse effects compared to other therapies. Despite the growing popularity of IFC therapy in various clinical settings, there have been few studies on its efficacy on intervertebral disc degeneration with sarcopenia patients. The aim of this study was to explore the effect of sarcopenia on IDD patients by comparing the characteristics of IDD patients with and without sarcopenia in the elderly population and their relationship. We also attempted to investigate the treatment effect of IFC on both patients.

## Methods

### Study design and participants

**Fig. 1:**
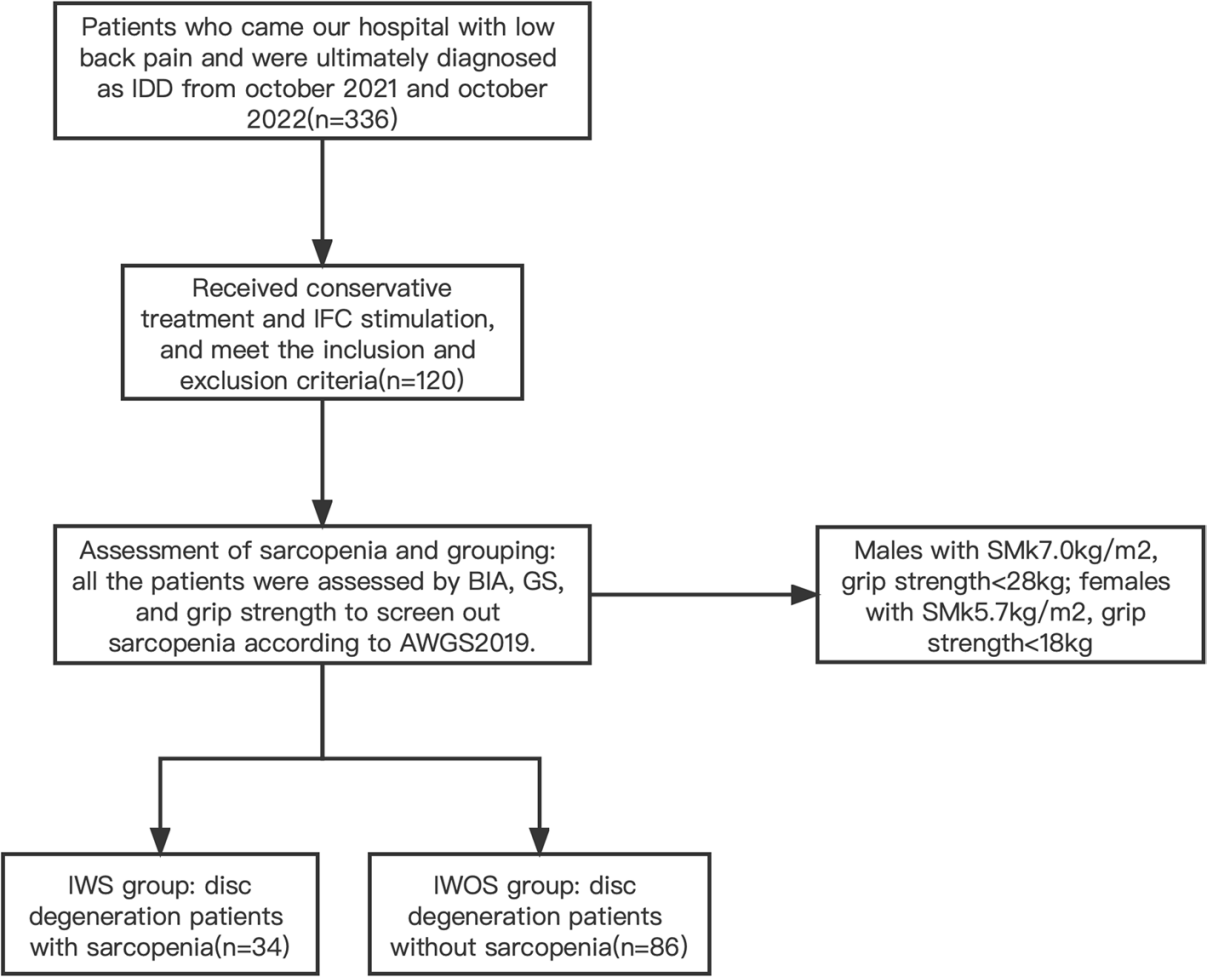
Overview of study flowchart. GS: Gait speed; SMI: Skeletal muscle index; BIA: Bioelectrical impedance analysis; IDD: Intervertebral disc degeneration; IFC*:* Interferential current; IWS: Intervertebral disc degeneration patients with sarcopenia; IWOS: Intervertebral disc degeneration patients without sarcopenia; AWGS2019: Asian Working Group for Sarcopenia 2019

This was a retrospective study including consecutive patients who came to our hospital with low back pain and were ultimately diagnosed (both clinical and radiological) as IDD. All included patients received conservative treatment and IFC stimulation between October 2021 and October 2022 (Fig. 1). The information of patients and indicators are collected through the hospital information system. This study was approved by the Institutional Research and Medical Ethics Committee of Second Xiangya Hospital of Central South University (Ethical review number: 744/2021). Written informed consent was obtained from each patient.

### Inclusion and exclusion criteria

Patients were included in the study if they conformed to the following criteria, (1) Patients diagnosed with IDD; (2) Magnetic resonance imaging (MRI) suggested disc degeneration; (3) The measurement indicators should include the following indicators: visual analogue scale (VAS), Oswestry dysfunction index (ODI), SARC-F, Skeletal muscle index (SMI), Gait speed (GS), grip strength and nerve conduction velocity (NCV); (4) Elderly patients whose age was over 60 years; (5) Received conservative treatment and IFC stimulation for 2 consecutive weeks. The exclusion criteria were, (1) patients with serious or complex medical illnesses (e.g.tumor, motor neuron diseases or cardiovascular disease); (2) who were taking hormones or physical therapy within 3 months.

### Assessment of sarcopenia and grouping

All the IDD patients who met the criteria were measured with bioelectrical impedance analysis (BIA) to obtain the SMI, which was calculated by dividing appendicular skeletal muscle mass (AMM) by body height in meters squared (kg/m^2^). The patient's grip strength and gait speed were also recorded. Participants were grouped according to the criteria of Asian Working Group for Sarcopenia 2019 (AWGS2019) [21]: (1) IDD with sarcopenia (IWS) group: disc degeneration patients with sarcopenia (Males with SMI < 7.0 kg/m^2^, grip strength < 28 kg; females with SMI < 5.7 kg/m^2^, grip strength < 18 kg); (2) IDD (IWOS) group: disc degeneration patients without sarcopenia.

### Therapeutic method

According to the medical records, all eligible patients received a consecutive two-week IFC therapy twice a day. Patients were asked to lay down in a prone position, with the low back area unclothed. The instruments (Bohua, BHE-200L, Japan) with four self-adhesive electrodes were used (9 × 5 cm) (ValuTrode®; Axelgaard, Fallbrook, CA). The electrodes of instrument were arranged in a cross-quadrupole arrangement (In the first channel, an electrode was positioned 3 cm to the right of L3's spinous process and 3 cm to the left of L5's; In the second channel, an electrode was positioned 3 cm to L3's left side and 3 cm to L5's right side. The following parameters were used: (1) a 4000 Hz carrier frequency; (2) a 65 Hz amplitude-modulated frequency; (3) a 1:1 swing pattern with a sweep frequency of 95 Hz. The intensity should be tolerated by the patient in terms of tingling, tremor, twitch and muscle contraction, which shall not exceed 70% of the maximum tolerable intensity with each stimulation lasting 20 min.

### Analysis variables

#### SARC-F

A simple and inexpensive screening method proposed by Malmstrom et al. in 2013 [22]. It consists of five items: muscle strength (ability to lift 10 pounds), walking ability (ability to walk 1 km), rise from a chair, climb stairs (ability to climb 10 steps) and falls (number of falls in past year). 2 points for each of these items, 10 points in total. Of the first four items, a score of 2 is given if the patient found it difficult or impossible to complete, 1 for somewhat difficult and 0 for easily doable. In the last item, 0 points for 0 falls, 1 point for 1 ~ 3 falls and 2 points for ≥ 4 falls. Total score ≥ 4 indicates increased risk of sarcopenia.

#### VAS

Participants were asked to rate their pain on visual analogue scale. A 10 cm line is drawn across the top of the paper, with 0 at one end of the line indicating no pain and 10 at the other end indicating severe pain.

#### ODI

The severity of lumbar dysfunction was evaluated according to Oswestry dysfunction index score [23], which consists of 10 items: pain level, self-care, lifting, walking, sitting, standing, sleep, sexual life, social life and travel. Each item is scored from 0 to 5, with the actual score/50 × 100% as the final score. The higher the score, the more pronounced the functional impairment.

### Functional measurements and radiological parameters

#### Grip strength

Grip strength was measured directly with a electronic hand dynamometer (CAMRY, MAX:90 kg, *d* = 100 g, EM101). During the test, the patient sits in a chair with the forearm resting on the armrest, then squeeze the grip dynamometer as tightly as possible within 3-5 s. The left and right hands are measured 3 times each, and the average reading of the 3 times is taken to represent the grip strength of the hand, and then the average grip strength of the left and right hands is taken to represent the patient's grip strength. The AWGS2019 recommended grip strength diagnostic thresholds are < 28 kg for men and < 18 kg for women [21].

#### Gait speed (GS)

6 m walk test was used to evaluate gait speed. During the test, the usual or comfortable pace is used as the standard, starting from the resting state, when the foot first touches the ground behind the line, the time taken to complete 6 m is recorded and the walking speed is obtained by calculating the ratio of distance to time, and measure at least 2 times, take the mean value as GS. A GS threshold of 1.0 m/s is recommended by the AWGS2019 standard [21].

#### Nerve conduction velocity (NCV)

NCV of the lower limbs was obtained through electrophysiological examination. The process was completed by professional technicians, and the NCV was obtained by calculating the ratio of distance (stimulation electrode to recording electrode) and time (electrical stimulation through stimulation electrode to recording electrode).

#### Standard cross-sectional area (SCSA)

The ImageJ (National Institutes of Health, Version 1.53, USA) was used to depict and calculate the cross-sectional area of the paravertebral muscles (psoas) in the L4/5 plane and the cross-sectional area of the corresponding disc, and the ratio of the two was SCSA [24].

#### Degree of fatty infiltration (DFF)

The ImageJ was used to depict and calculate fat infiltration area of the paravertebral muscles (psoas) and the area of the psoas in the L4/5. The ratio of the two is DFF [24].

#### SMI

Bioelectrical impedance analysis (Inbody, Inbody760, Korea) was used to obtain skeletal muscle index. During the process, the test is carried out by applying a weak current or voltage to the subject through surface electrodes, thereby detecting the electrical impedance and changes, and ultimately obtaining information about AMM. The ratio of AMM to height squared is the SMI. The test is performed on an empty stomach to reduce the interference of water with the results. According to AWGS2019, males < 7.0 kg/m^2^ and females < 5.7 kg/m^2^ can be diagnosed with low skeletal muscle mass measured by BIA [21].

#### Pfirrmann grading for evaluating lumbar disc degeneration [25]

Grade I: The disc's structure is homogenous, and it has a typical disc height and a bright, hyperintense white signal. Grade II: The disc structure is inhomogeneous, with a white signal that is hyperintense. The nucleus and anulus are clearly distinguished, and the disc height is typical, with or without horizontal gray bands. Grade III: The disc structure is inhomogeneous, with a gray signal intensity that is intermediate. The disc height is normal or slightly diminished, and the differentiation between nucleus and anulus is uncertain. Grade IV: The disc structure is inhomogeneous, with a signal intensity that is hypointense dark gray. The disc height is normal or considerably diminished, and the difference between nucleus and anulus is obliterated. Grade V: The disc structure is inhomogeneous, and the black signal intensity is hypointense. The disc space is compacted, and the difference between nucleus and anulus is gone. T2-weighted midsagittal rapid spin-echo images are used to grade.

### Statistical analysis

SPSS 21.0 (IBM, Version 21.0, USA) was applied to analyze the data, and the measurement data were expressed as mean ± standard deviation (x ± s). Data normality and homogeneity of variance was evaluated by Kolmogorov–Smirnov test and Levene test, respectively, and all the outcomes had normal distribution. Between-group differences in baseline characteristics were evaluated by one way ANOVA (parametric data) and Kruskal–Wallis (non-parametric data). Categorical data are expressed as frequencies (percentages), with between-group differences being analyzed using Fisher’s exact test. Two independent sample t-test is used for the comparison between the two groups, and paired t-test is used for the comparison before and after treatment in a single group. Cohen’s *d* test was used to calculate the effect size (ES) and it was classified as small (0.0–0.2), moderate (0.3–0.5), or large (⩾0.6). Count data were expressed as number of cases, and the χ2 test was used for comparison between groups. Spearman's correlation analysis of SCSA, DFF and Pfirrmann grading was performed. The difference was considered statistically significant at *P* < 0.05.

## Results

**Fig. 2:**
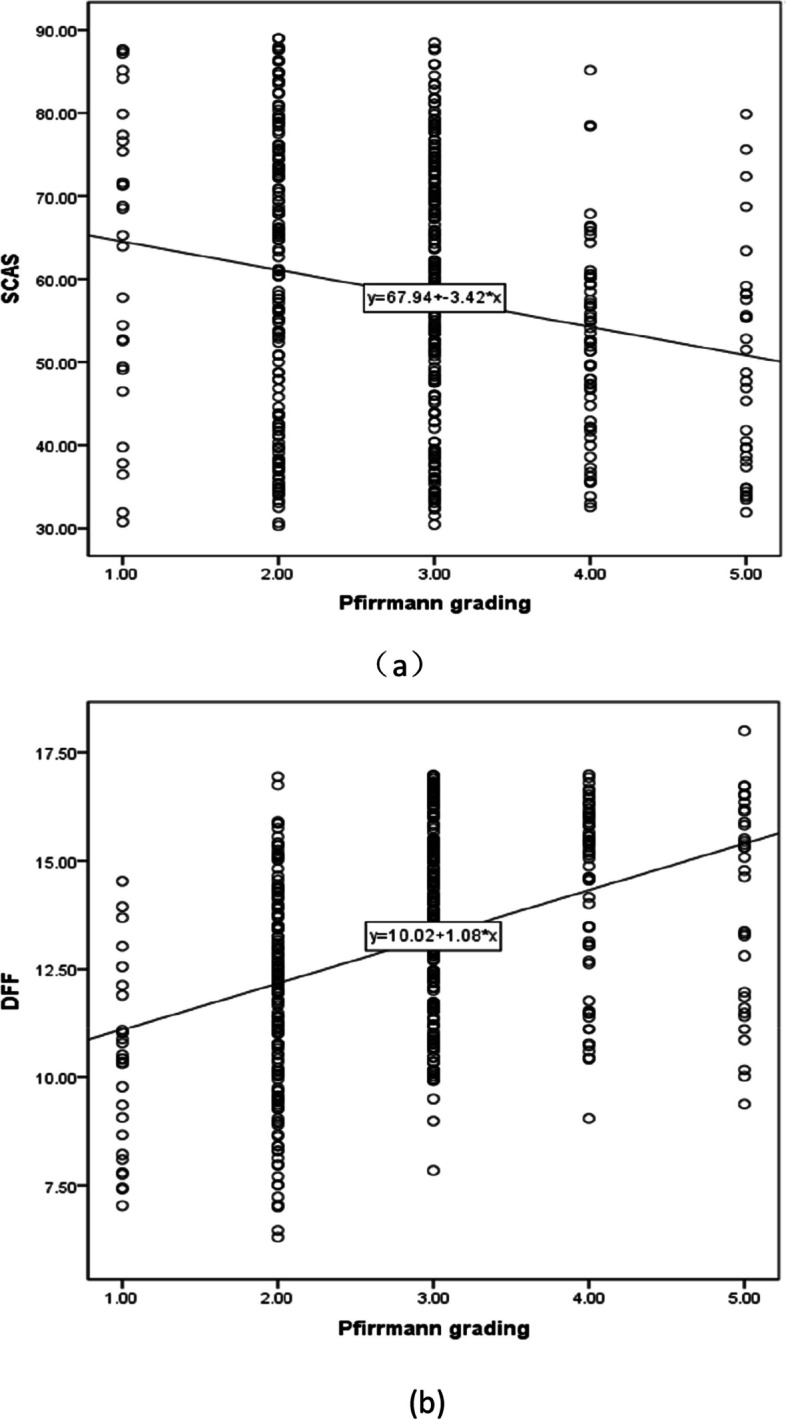
Correlation analysis. **a** Correlation analysis of Pfirrmann grading and SCSA of included patients; **b** Correlation analysis of Pfirrmann grading and DFF of included patients. SCSA: Standard cross-sectional area; DFF: Degree of fatty infiltration

1. The prevalence of sarcopenia in IDD patients was 28.3%. The difference of VAS, ODI, disc Pfirrmann grading, SCSA, DFF and NCV between the two groups were statistically significant before intervention (*P* < 0.05), the SCSA and DFF of paraspinal muscles in both groups correlated with the degree of IDD (*r* = -0.216, *r* = 0.430; *r* = -0.152, *r* = 0.499; *r* = -0.137, *r* = 0.435; *r* = -0.236, *r* = 0.346) (Table 1; Fig. 2a, b);
2. The improvement of SMI, GS, grip strength, VAS, SARC-F and ODI in IWS group was statistically significant after intervention (*p* < 0.05) (Table 2), and the ones of SMI, GS, grip strength, VAS and ODI in IWOS group were also statistically significant after intervention (*P* < 0.05) (Table 2); while the differences of disc Pfirrmann grading, SCSA, DFF and NCV in both groups were not statistical significant (*P* > 0.05) (Table 2).
3. The improvements of grip strength, GS, ODI, and SARC-F were significantly greater in the IWS group than in the IWOS group after IFC therapy, and the differences were statistically significant (*P* < 0.05) (Table 3).

**Table 1:** Comparison and correlations in SCSA (mm2) and DFF (%) for paravertebral muscles of different Pfirrmann gradings (x ± s)

Pfirrmann grading	Number of discs (240)	Paravertebral muscle (IWS before)		Paravertebral muscle (IWS after)		Paravertebral muscle (IWOS before)		Paravertebral muscle (IWOS after)	
		SCAS	DFF	SCAS	DFF	SCAS	DFF	SCAS	DFF
I	14	60.42 ± 14.69	9.16 ± 2.41	59.05 ± 18.41	10.00 ± 2.25	67.44 ± 12.58	11.46 ± 1.76	66.73 ± 17.26	10.65 ± 1.95
II	78	56.42 ± 15.28	12.41 ± 2.77	56.10 ± 17.78	11.50 ± 2.45	61.57 ± 14.93	12.17 ± 1.91	63.78 ± 15.77	12.23 ± 2.25
III	101	52.53 ± 13.91	14.48 ± 2.36	54.69 ± 15.05	14.74 ± 2.08	59.51 ± 15.96	13.32 ± 1.87	64.97 ± 13.75	12.92 ± 1.83
IV	31	48.86 ± 12.20	15.17 ± 1.79	50.01 ± 12.43	14.56 ± 2.30	59.89 ± 14.43	14.06 ± 1.61	53.66 ± 10.42	13.96 ± 1.88
V	16	48.83 ± 12.83	14.04 ± 2.51	46.30 ± 14.49	14.10 ± 2.73	55.15 ± 14.67	14.40 ± 1.91	51.54 ± 12.13	13.96 ± 2.09
r		-0.216	0.430	-0.152	0.499	-0.137	0.435	-0.236	0.346
p		0.001	0.000	0.019	0.000	0.034	0.000	0.000	0.000

*SCSA* Standard cross-sectional area, *DFF* Degree of fatty infiltration, *IFC* Interferential current, *IWS* Intervertebral disc degeneration patients with sarcopenia, *IWOS* Intervertebral disc degeneration patients without sarcopenia

^*^P < 0.05, statistically significant differences. Before IFC stimulation: *r* = -0.216, *r* = 0.430; *r* = -0.137, *r* = 0.435; After IFC stimulation: *r* = -0.152, r = 0.499; *r* = -0.236, *r* = 0.346

**Table 2:** Index and comparison of IDD patients with sarcopenia and IDD without sarcopenia before and after IFC therapy (x ± s)

	IWS group (before)	IWS group (after)	IWOS group (before)	IWOS group (after)	*P*	ES	*P*	ES	*P*	ES
					IWS(before) vs IWOS(before)		IWS(before) vs IWS(after)		IWOS(before) vs IWOS(after)	
Number of cases	34	34	86	86						
Grip strength	17.33 ± 3.36	23.64 ± 4.42	27.80 ± 2.82	28.56 ± 2.72			0.000	1.607	0.003	0.274
GS	0.88 ± 0.08	1.06 ± 0.10	1.21 ± 0.15	1.37 ± 0.12			0.000	1.988	0.000	1.178
SMI	5.89 ± 0.68	6.38 ± 0.86	7.22 ± 0.83	7.57 ± 0.58			0.002	0.632	0.000	0.489
SARC-F	5.50 ± 1.24	3.50 ± 1.11	2.44 ± 1.01	2.34 ± 0.97			0.000	1.700	0.479	0.101
SCSA	53.53 ± 14.39	54.24 ± 16.02	60.40 ± 15.24	62.33 ± 14.85	0.000	0.463	0.591	0.047	0.124	0.128
DFF	13.56 ± 2.86	13.35 ± 2.82	13.00 ± 2.01	12.77 ± 2.15	0.000	0.227	0.332	0.074	0.161	0.111
VAS	7.38 ± 1.10	2.79 ± 1.59	6.09 ± 1.81	1.24 ± 0.91	0.000	0.861	0.000	3.357	0.000	3.386
ODI	20.44 ± 3.49	14.32 ± 2.43	13.55 ± 3.06	11.88 ± 2.53	0.000	2.099	0.000	2.035	0.000	0.595
NVC	41.34 ± 3.52	42.86 ± 4.81	45.10 ± 4.83	45.77 ± 4.21	0.001	0.890	0.153	0.361	0.281	0.148

All the comparisons were performed between two groups, the detailed information was indicated in the table (two-tailed, Student’s* t* test), *P < 0.0125, statistically significant differences (Bonferroni Correction, α_adjusted_ = α/n, α = 0.05, *n* = 4). Effect size was presented by Cohen’s *d*. Cohen’s *d*: 0–0.2 small; 0.3–0.5 moderate; ≥ 0.6 large

*GS* Gait speed, *SMI* Skeletal muscle index, *SARC-F* S (Strength), *A* (Assistance in walking), *R* (Rise from a chair), *C* (Climb stairs), *F* (Falls), *SCSA* Standard cross-sectional area, *DFF* Degree of fatty infiltration, *VAS* Visual analogue scale, *ODI* Oswestry dysfunction index, *NCV* Nerve conduction velocity, *IDD* Intervertebral disc degeneration, *IFC* Interferential current, *IWS* Intervertebral disc degeneration patients with sarcopenia, *IWOS* Intervertebral disc degeneration patients without sarcopenia, *ES* Effect size

**Table 3:** Comparison of the difference in improvement of observed indexes between IDD patients with sarcopenia and without sarcopenia after IFC therapy (x ± s)

	IWS	IWOS	*P*	ES
Number of cases	34	86		
Grip strength	6.31 ± 4.22	0.76 ± 2.32	0.000	1.630
GS	0.25 ± 0.15	0.16 ± 0.16	0.003	0.580
SMI	0.49 ± 0.86	0.35 ± 0.81	0.431	0.168
SARC-F	-2.00 ± 1.44	-0.10 ± 1.36	0.000	1.357
SCAS	1.93 ± 23.27	-2.57 ± 20.72	0.303	0.204
DFF	0.05 ± 3.15	-0.36 ± 3.51	0.561	0.123
VAS	-4.59 ± 1.99	-4.85 ± 1.91	0.507	0.133
ODI	-6.12 ± 4.11	-1.66 ± 4.01	0.000	1.098
NVC	1.51 ± 6.04	0.67 ± 5.71	0.474	0.143

After IFC stimulation, the improvement of grip strength, GS, ODI and SARC-F in IWS group were greater than IWOS (*P* < 0.05), whereas there was no significance in improvement degree of other indicators between the two (*P* > 0.05); *P < 0.05, statistically significant differences. All the comparisons were performed between two groups, the detailed information was indicated in the table (two-tailed, Student’s* t* test). Effect size was presented by Cohen’s *d*. Cohen’s *d*: 0–0.2 small; 0.3–0.5 moderate; ≥ 0.6 large

*GS* Gait speed, *SMI* Skeletal muscle index, *SARC-F* S (Strength), *A* (Assistance in walking), *R* (Rise from a chair), *C* (Climb stairs), *F* (Falls), *SCSA* Standard cross-sectional area, *DFF* Degree of fatty infiltration, *VAS* Visual analogue scale, *ODI* Oswestry dysfunction index, *NCV* Nerve conduction velocity, *IDD* Intervertebral disc degeneration, *IFC* Interferential current, *IWS* Intervertebral disc degeneration patients with sarcopenia, *IWOS* Intervertebral disc degeneration patients without sarcopenia, *ES* Effect size

## Discussion

Patients with IDD often suffer from low back pain and neurological deficit (e.g. numbness of legs, walking dysfunction, urinary and fecal disorders) which drastically affect their quality of life. Previous studies have suggested an association between degenerative spinal diseases and skeletal muscle, the average area of paravertebral muscles in lumbar disc herniation (LDH) patients is smaller than that of healthy people, and the reduction is more significantly in those whose unilateral symptoms are the main manifestation [26–31]. Eguchi et al. demonstrated that sarcopenia is associated with spinal deformities such as degenerative lumbar scoliosis and lumbar spinal stenosis in an elderly female patients [32]. Zotti et al. suggested that preoperative atrophy and reduction in cross-sectional area of the multifidus muscle are associated with postoperative complications after lumbar decompression surgery [33]. In our study, the prevalence of sarcopenia in IDD was 28.3%, much higher than the number (9%) in general population, and also consistent with previous studies suggesting 25% in LSS [34, 35].

### Interaction between IDD and sarcopenia

#### Sarcopenia may be promoted by IDD

Our study indicated that sarcopenia was more common in IDD patients than normal, and that SCSA and DFF in paraspinal muscles were significantly worse in IWS patients compared to IWOS patients. In addition, the SCSA and DFF were correlated with the degree of disc degeneration. We deduced that IDD may promote or contribute to the occurrence and development of sarcopenia to some extent. On one hand, IDD patients often suffer from low back pain or/and leg pain due to the compression, irritation or inflammation of nerve root, leading to chronic pain and lower extremity dysfunction, and patients with long-term activity restriction are prone to sarcopenia [36, 37]. On the other hand, the process may be accompanied by secondary changes in the structure of the spine, which in the long term leads to decompensation of the skeletal muscles (mainly paraspinal muscles), increased pain and activity dysfunction [8, 38, 39].

#### Sarcopenia may worsen the symptoms of IDD patients

The progression of sarcopenia could result in a decrease in the muscle mass and strength, thus reduced stability of the discs or intervertebral joints, and therefore promotes degeneration. We found that in addition to poorer SMI, GS, and grip strength, IWS patients had worse SCAS, DFF, and NCV than IWOS patients (*P* < 0.05; Table 2). Meanwhile, the neurological function of sarcopenic patients may be impaired, thus worsen the symptoms of the IDD, which also reflected in our study that in addition to SARC-F, the VAS and ODI in IWS group were higher than IWOS group (*P* < 0.05; Table 2). The above explains why IWS patients exhibit more severe pain, poorer neurological function and worse activity status. Previous studies have found that degeneration of the paravertebral muscles may play an important role in the sagittal or coronal balance of spine, meaning that the biologic load on the discs increases when spinal stability decreases, eventually leading to IDD or even LDH, causing nerve root compression, low back pain, and lower extremity dysfunction [40–42]. Our study also confirmed that psoas degeneration was associated with the severity of disc degeneration, and sarcopenic patients with significant psoas degeneration have more severe pain, worse mobility and function (Table 1). Furthermore, it has been found that there is a decrease in the number of Muscle Stem Cells (MSCs) or Satellite Cells (SCs) in sarcopenia patients, which are essential for the regenerative and repair capacity of skeletal muscle [43, 44]. As a result, sarcopenic patients have poorer muscle mass, strength, and physical performance. This may also explain why clinical symptoms and complications are more pronounced in IDD patients who have sarcopenia. Although the role of stem cells in the genesis of sarcopenia is still under discussion [45], keeping a healthy satellite cell population or exogenously introducing a regenerative progenitor population to aging muscle has the potential to reverse the acquired impairments brought on by sarcopenia.

#### IFC is effective in both IWS and IWOS patients

A lot of IWS patients may not tolerate or even refuse exercise therapy because of pain, despite the fact that resistance and aerobic exercise are suggested as the best early treatment for sarcopenia. Additionally, “bad” emotion may also prevent patients from accepting higher-intensity exercise. Physiotherapy showed increasing prospects in the treatment of skeletal muscle disorders which recent studies pointed out [13, 46–49], and our results also revealed that IFC is not only effective in IDD patients, but also be effective in sarcopenia.

Interferential current therapy is widely used by different clinicians around the world and it is one of the electrotherapy techniques for the management of musculoskeletal disorders and improvement of somatic functions [49–53]. IFC is the application of an alternating medium frequency current (4000 Hz) amplitude modulated at a low frequency (0-250 Hz) [13, 54]. At this frequency, IFC is claimed to have better penetration to deeper tissue while overcoming the problem of skin impedance without causing discomfort [18, 55, 56]. When applied to specific tissues, IFC improves physical function and quality of life [13] by accelerating the uptake of inflammation around nerve roots and pain-related chemokines of the degenerative disc [57, 58]. On the basis of previous studies, our study confirmed the effectiveness of IFC in IWOS patients (Table 2), that is, the body function (GS, gait speed, ODI) and pain (VAS) of IWOS patients were significantly improved after IFC therapy.

Since the discovery of electricity, it has been known that electricity stimulates nerve fibers and induce muscle contractions during physiotherapy, and low and medium waveforms can be produced by varying the frequency of the electrical current. The effect of electrical stimulation on muscles enhances local metabolism, increases neuromuscular excitability and motor neurons, promotes the growth and germination of axons and neuromuscular junction regeneration [59], thus repairs and rebuilds the damaged or atrophied denervated skeletal muscles, and improves muscle quality and strength [15, 60–62]. Blickenstorfer and Kampe reported that the application of electrical stimulation to some skeletal muscles could induce brain plasticity of the pain-associated sensorimotor cortex. It changes the polarization state of nerve cell membrane by activating the region, altering neuronal excitability, generating action potential, causing muscle contraction and stimulating passive movements, thus enhances muscle strength, and mitigates the effects of skeletal muscle recession [63, 64]. Nonetheless, IWS group did not exhibit significant improvement in nerve conduction after IFC therapy in our research. Although the nerve structures may be healed, neurological function was not entirely restored. This may be due to the slower pace of nerve growth and the fact that the length of the IFC in the study was less than the period needed for nerve repair.

Despite no increase in neurological function, IFC was found significantly enhanced muscle function in our study. It is said that IFC induces involuntary contraction of skeletal muscle to promote its anabolism to increase fibronectin content in muscle and maintain or improve skeletal muscle mass and strength [46, 65–70]. An in-depth study found that it may reduce the concentration of calcium in mitochondria and calcium overload by increasing the activity of Na^+^-K^+^-ATPase and Ca^2+^-Mg^2+^-ATPase, thus stimulates oxidative phosphorylation, which plays a central role in reducing fatigue and improving muscle function [71, 72]. This conclusion was also supported by our investigation, where skeletal muscle index, grip strength, gait speed, ODI and SARC-F were improved significantly after intervention in IWS group (Table 2). At the same time, the skeletal muscle index, grip strength, gait speed, and ODI of the IWOS group also showed significant improvement after IFC therapy (Table 2). Among them, the skeletal muscle index intuitively reflects the mass of the skeletal muscles of the whole body, grip strength reflects skeletal muscle strength, while GS, ODI and SARC-F strongly reflect skeletal muscle-based physical functions. So the significant changes of the above indexes in the two groups after IFC therapy confirm the improvement of muscle function and the effectiveness of IFC therapy for sarcopenia. Interestingly, in IWOS patients, the SARC-F, which is an important scale evaluating physical function for screening sarcopenia, did not display statistical differences after the IFC therapy (Table 2). Therefore, the differences between before and after therapy of the two groups suggested that there was a close connection between sarcopenia and physical function, meaning that skeletal muscle function may play an important role in ensuring somatic function. Previous studies have made similar conclusions, explaining that deep muscles contribute to joint stability and help maintain posture; thus, dysfunction of deep muscles is significantly associated with increased risk of falls and decreased walking function [73–75].

A closer analysis revealed that the improvements of grip strength, GS, ODI, and SARC-F were significantly greater in IWS patients than IWOS patients after IFC therapy, and the differences were statistically significant (*P* < 0.05) (Table 3). It implied that IFC stimulation significantly improved skeletal muscle strength, function and body performance in sarcopenic patients although no differences were found in improving skeletal muscle quality, but this could still indicate that IFC has a certain effect on sarcopenia. Unfortunately, we were unable to find statistical differences in SCAS and DFF before and after IFC therapy between the two groups (Table 2), which may be caused by the fact that only the SCAS and DFF of the paravertebral muscles at the L4/5 plane were examined in this study. In addition, we cannot deny the impact of shorter IFC treatment cycles and follow-up times. All those may lead to the inability to observe significant statistical differences in SCAS and DFF of the two groups before and after IFC therapy. Therefore, the observation of the efficacy of these two measures requires longer periods and further studies. What’s more, the exact mechanism of action of IFC therapy is not known and the means to identify it remain to be developed.

### Limitation

The research results reported here should be considered in the light of some limitations. Although there was no significant difference in age, gender, BMI, smoking, and osteoporosis between the two groups (Table 4), all the patients in our study are Asian, so the influence of race was exclueded. However, nutrition, inflammation and living habit such as diet and sleep, which have impact on sarcopenia could not be standardized. Apart from this, this retrospective study failed to indicate the optimal cycle and frequency of IFC for IDD with sarcopenia patients due to socio-economic factors and patient-specific circumstances (e.g., disparities in accessibility to healthcare, and educational and patient's compliance), although this may have an important impact on neurological recovery and skeletal muscle growth. Additionally, only L4/5 paravertebral muscles were examined in our study, and errors in the measurement process can not be avoided totally. Meanwhile, the long-term effects of IFC on sarcopenia or IDD remain unknown due to follow-up limitations. These are the sources of limitations and biases in the study. Therefore, in future studies, it may be necessary to further investigate the long-term effects of interferential currents, conduct randomized controlled trials, or explore other potential interventions for disc degeneration and sarcopenia.

**Table 4:** Baseline clinical characteristics of IDD patients with and without sarcopenia

Characteristic	IWS group (*n* = 34)	IWOS group (*n* = 86)	*P*
Age	68.62 ± 2.35	68.08 ± 1.89	0.194^a^
Sex(female/male)	16/18	41/45	0.951^b^
BMI(kg/m^2^)	23.1 ± 2.6	22.7 ± 2.5	0.413^a^
Smokers, n (%)	11(32.4)	26(30.2)	0.829^b^
Osteoporosis, n (%)	19(55.9)	39(45.3)	0.306^b^

Values are presented as mean ± SD or count

*IWS* Intervertebral disc degeneration (IDD) patients with sarcopenia, *IWOS* Intervertebral disc degeneration (IDD) patients without sarcopenia, *BMI* Body mass index

^a^Data that was normally distributed and analyzed with ANOVA

^b^Statistical analysis was performed using Fisher’s Exact test

*P* < 0.05, statistically significant differences

## Conclusion

The prevalence of sarcopenia was high in IDD patients, and the degree of paravertebral muscles degeneration was correlated with the degree of IDD. Compared with IWOS patients, IWS patients have more severe pain, poorer mobility, and worse neurological function. After IFC therapy, both IWS and IWOS patients had effective symptom relief. The improvement of skeletal muscle strength and function in IWS patients was significantly greater. Therefore, IFC stimulation is effective in elderly IDD patients with or without sarcopenia.

## Data Availability

The datasets analyzed during the current study are available from the corresponding author on reasonable request.

## Abbreviations

IDD: Intervertebral disc degeneration
LBP: Lower back pain
LSS: Lumbar spinal stenosis
IFC: Interferential currents
IWS: Intervertebral disc degeneration with sarcopenia
IWOS: Intervertebral disc degeneration without sarcopenia
MRI: Magnetic resonance imaging
VAS: Visual analogue scale
ODI: Oswestry dysfunction index
SMI: Skeletal muscle index
GS: Gait speed
NCV: Nerve conduction velocity
BIA: Bioelectrical impedance analysis
AMM: Appendicular skeletal muscle mass
AWGS2019: Asian Working Group for Sarcopenia 2019
SCSA: Standard cross-sectional area
DFF: Degree of fatty infiltration
LDH: Lumbar disc herniation
MSCs: Muscle Stem Cells
SCs: Satelite Cells
